# Deep learning in oral cancer- a systematic review

**DOI:** 10.1186/s12903-024-03993-5

**Published:** 2024-02-10

**Authors:** Kritsasith Warin, Siriwan Suebnukarn

**Affiliations:** Faculty of Dentistry, Thammasat University, Pathum Thani, Thailand

**Keywords:** Artificial intelligence, Deep learning, Neural network, Oral cancer, Systematic review

## Abstract

**Background:**

Oral cancer is a life-threatening malignancy, which affects the survival rate and quality of life of patients. The aim of this systematic review was to review deep learning (DL) studies in the diagnosis and prognostic prediction of oral cancer.

**Methods:**

This systematic review was conducted following the PRISMA guidelines. Databases (Medline via PubMed, Google Scholar, Scopus) were searched for relevant studies, from January 2000 to June 2023.

**Results:**

Fifty-four qualified for inclusion, including diagnostic (*n* = 51), and prognostic prediction (*n* = 3). Thirteen studies showed a low risk of biases in all domains, and 40 studies low risk for concerns regarding applicability. The performance of DL models was reported of the accuracy of 85.0–100%, F1-score of 79.31 - 89.0%, Dice coefficient index of 76.0 - 96.3% and Concordance index of 0.78–0.95 for classification, object detection, segmentation, and prognostic prediction, respectively. The pooled diagnostic odds ratios were 2549.08 (95% CI 410.77–4687.39) for classification studies.

**Conclusions:**

The number of DL studies in oral cancer is increasing, with a diverse type of architectures. The reported accuracy showed promising DL performance in studies of oral cancer and appeared to have potential utility in improving informed clinical decision-making of oral cancer.

**Supplementary Information:**

The online version contains supplementary material available at 10.1186/s12903-024-03993-5.

## Background

Oral cancer is one of the major causes of death globally, the 17th most common worldwide and the 11th most common in Asia. According to the World Health Organization, more than 370,000 new cases of oral cancer were reported and caused over 170,000 deaths in 2020 [1]. There are various types of oral cancer depending on its origin (carcinoma and sarcoma), but the most common type is oral squamous cell carcinoma (OSCC), which is mostly transformed from oral potentially malignant disorders (OPMDs). The definitive gold standard diagnostic tool of oral cancer and OPMDs is surgical biopsy and histopathologic evaluation [2, 3]. The treatment modalities for oral cancer were surgery, radiotherapy, and chemotherapy either alone or in combination, which is generally determined according to the stage of the disease. The treatment outcomes, especially in advanced stages, have resulted in high morbidity, affecting the masticatory function, facial esthetics, and quality of life of oral cancer patients [2]. Currently, advances in oral cancer treatment have not improved the prognosis of oral cancer over the past decade [4]. Oral cancer prognosis has been based on cancer staging [5], which decreases significantly in advanced stages compared to early stages of oral cancer or in the stage of OPMDs. Therefore, the early diagnosis of oral cancer is the crucial step to increase the survival rate of oral cancer patients.

Deep learning (DL), a subset of artificial intelligence (AI), is built based on neural networks, which are biologically inspired programming algorithms that have the ability to learn complex representations to improve pattern recognition from raw data [6]. These algorithms are composed of multiple layers, which transform input data (such as medical images) into outputs (such as diagnostic or prognostic recommendations) while automatically learning higher-level features [6, 7]. DL has been proven capable of analyzing complex data and is widely applied in the medical field, including diagnostics, detecting abnormalities in medical images, etc. [7]. Integrating DL technology into routine clinical practice relies on achieving diagnostic accuracy that is not inferior to professional healthcare. In addition, it must provide other benefits, such as speed, efficiency, reduced cost, enhanced accessibility, and ethical conduct [8].

Nowadays, DL research in oral cancer is highly dynamic and keeps increasing due to its feasibility and many advantages to improve the cancer survival rate in the aspect of detection, prevention, and prognostic prediction [8–10]. There are studies that developed a mobile phone-based application for the oral cancer screening as an alternative method for early detection of oral cancer with a high accuracy to distinguish oral lesions from clinically suspicious lesions, which showed the potential of the application of computer-assisted visualization in the clinical practice [11, 12]. Application of DL to oral cancer data can assist clinicians in the diagnosis, detection, and prognostic prediction of oral cancer in clinical practice for early diagnosis and selection of the most appropriate treatment to increase the survival rate of patients with oral cancer.

There have been some previous systematic reviews on AI and machine learning in oral cancer [13, 14]. This study, therefore, mainly focused on the application of DL, which is the neural network-based architecture that has an ability to learn complex features, on oral cancer data. The main objective of this study is to systematically analyze evaluation studies of the application of DL in oral cancer data to aid in the diagnosis, detection, and prognostic prediction of oral cancer, and compare their results regarding the reported performance measures. In addition, this study further aimed to synthesize the results and assess the robustness of the body of evidence of DL-based diagnostic and prognostic predictive models on oral cancer data.

## Methods

This is a systematic review of diagnostic and prognostic prediction studies. Reporting of this study follows the PRISMA guideline [15]. The study protocol was registered at the international prospective register of systematic reviews (PROSPERO) (CRD42023425992).

### Inclusion criteria and exclusion criteria

The eligible studies must have evaluated the diagnostic or prognostic significance of oral cancer using DL algorithms. Publications were selected for review if they satisfied the following inclusion criteria: full texts available in English language; studies using DL (of any class) to provide diagnostic and prognostic prediction information of oral cancer and OPMDs; studies providing outcomes of model performance (diagnostic and prognostic prediction accuracy) and/or compared to a human diagnostic performance. For DL-based diagnostic studies in clinical and radiographic images (classification, detection, or segmentation), ground truth of captured images was identified by histopathologic result as the gold standard diagnosis of oral cancer and OPMDs.

**Fig. 1:**
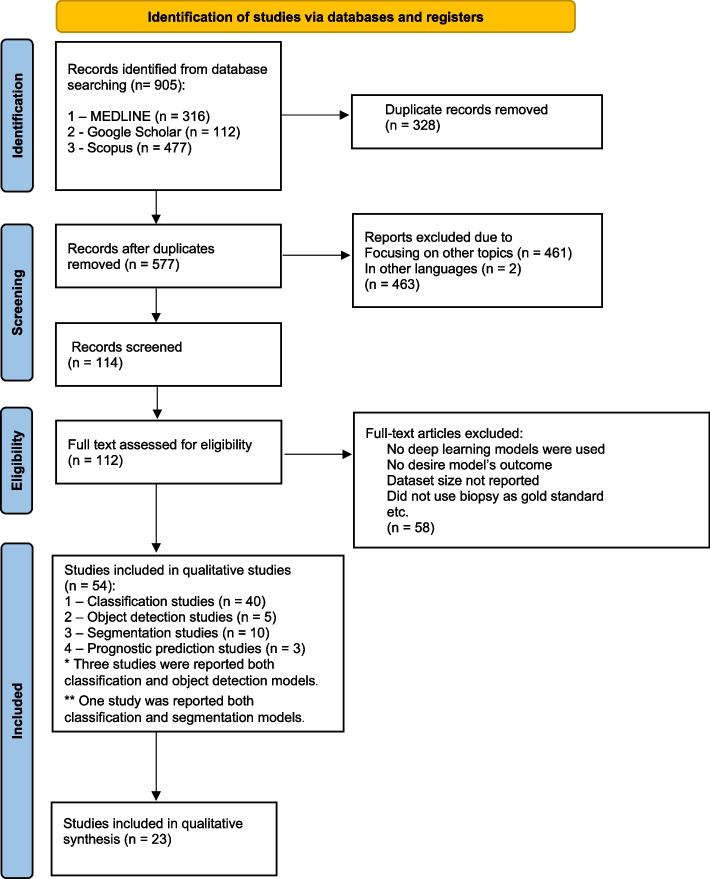
Flow diagram of search methodology and literature selection process

Studies with the following criteria were excluded: studies where ground truth of DL-based diagnostic studies was not explicitly confirmed; studies of machine learning (ML) applications without DL algorithms; studies without sufficient details on the data used for training and testing (e.g., dataset size, data modality, etc.); studies without a clear explanation of the DL model; studies that examined DL applications for normal oral mucosa, oral lesions (without cancer or OPMDs), periodontal disease, or dental caries, DNA and RNA microarray genes, proteomics, fluorescence spectroscopy, and genetic programming; articles in languages other than English. The details of the inclusion and exclusion criteria are presented in Fig. 1.

### Information sources and search

An electronic search was conducted in the following electronic databases up to 14th June 2023: Medline via PubMed, Google Scholar, and Scopus. The search was conducted from January 2000 through June 2023. Each database was searched with adapted keywords. The search query for each database is described in Table 1.

**Table 1:** The results of the electronic search in the various databases

Database	Keywords	Results	Date
Medline via PubMed	((artificial intelligence [MeSH]) OR “artificial intelligence” OR (machine learning [MeSH]) OR “machine learning” OR (deep learning [MeSH]) OR “deep learning” OR “neural network” OR “computer vision”) AND (“oral cancer” OR “oral squamous cell carcinoma” OR “oral potentially malignant disorder” OR “oral precancerous” OR (mouth neoplasms [MeSH]))	316	14 June 2023
Google Scholar	allintitle:(“artificial intelligence” OR “machine learning” OR “deep learning” OR “neural network” OR “computer vision”) AND (“oral cancer” OR “oral squamous cell carcinoma” OR “oral potentially malignant disorders” OR “oral precancerous”)	112	14 June 2023
Scopus	(“artificial intelligence” OR “machine learning” OR “deep learning” OR “neural network” OR “computer vision”) AND (“oral cancer” OR “oral squamous cell carcinoma” OR “oral potentially malignant disorder” OR “oral precancerous”)	477	14 June 2023

### Study selection

For managing the citations, Endnote 20 (Clarivate, Philadelphia, USA) was used. Two independent reviewers performed title and abstract screening after removing duplicate papers (K.W. and S.S.). Then, the reviewers evaluated full texts of eligible studies based on inclusion and exclusion criteria. Any disagreements or discrepancies were resolved by discussion and consensus of the two reviewers.

### Data collection and extraction

Two reviewers (K.W. and S.S.) independently collected data from the included studies. Any disagreements or discrepancies were resolved by discussion and consensus of the two reviewers. The following data items were extracted: bibliographic details (name of authors, the year of publication and country), data modality, dataset size (train/valid/test, if given), augmentation, DL algorithms examined in the study, the definition of the study objective (diagnostic or prognostic), ground truth identification and annotation and task (classification, detection, segmentation) in the DL based diagnostic study, hyperparameters of the DL models, hardware used, performance metrics reported, including precision, recall, accuracy, sensitivity, specificity, F1-score, average precision (AP), Dice index, area under receiving operating characteristics curve (AUC), Concordance index (c-index) and Integrated Brier score (IBS). If more than one model was used, this study only reported on the best performance model.

### Risk of bias and applicability

The methodological quality of the included studies was evaluated using the Quality Assessment of Diagnostic Accuracy Studies (QUADAS-2) tool [16] for risk of bias assessment. The QUADAS-2 checklist consists of four risks of bias domains, including patient selection, index test, reference standard, and flow and timing. Any disagreements between the two reviewers were resolved by discussion and consensus. Some questions were slightly modified to specifically assess studies on DL [17]. In “patient selection”, limited information about the presented dataset as well as unclear data allocation strategies were considered to indicate a high risk of bias. For “index test”, insufficient information on model construction, including hyperparameters, and lack of model robustness analyzes were considered to indicate high risk of bias. For “reference standard”, the lack of information on the definition of the reference standard and the use of a single examiner to establish the reference test were considered to indicate a high risk of bias. Finally, in “flow and timing”, the indicators used different reference standards in the same study and inappropriate intervals between the index test and the reference standard. Details of the modified QUADAS-2 tool are provided in the supplemental information (Table 1S).

### Statistical analysis

All statistical analyses were performed using R software, version 3.6.3 (Vienna, Austria) and IBM SPSS Statistics version 26. Because a few studies reported the number of true positives (TP), true negatives (TN), false positives (FP) and false negatives (FN). This study used the diagnostic odds ratios (DOR) as pooled outcome from the reported sensitivity and specificity to determine the diagnostic accuracy of the deep learning system [18], calculated as follows:

$$DOR=\frac{Sensitivity\times Specificity}{\left(1- Sensitivity\right)\times \left(1- Specificity\right)}$$

## Results

### Study selection and study characteristics

The search results and process of selecting articles are shown in Fig. 1. After the literature search, a total of 905 articles were identified. Articles were excluded for the following reasons: studies that were duplicated (*n* = 328), studies focusing on other topics (*n* = 461), and studies that were not written in English language (*n* = 2). A total of 112 studies were assessed in full text. Fifty-eight of these studies, including studies that did not use DL models, studies that did not report the desired outcomes and dataset size, and studies on clinical images that did not use biopsy as the gold standard, were excluded after full text assessment.

### Characteristics of relevant studies

The individual studies are summarized in Tables 2, 3, 4 and 5 with each table showing studies using DL in diagnostic studies, including classification (Table 2), object detection (Table 3), segmentation (Table 4), and prognostic prediction studies (Table 5).

**Table 2:** Summary of findings in the selected diagnostic studies (classification)

No	Author, Year (Ref)	Country	Data Modality (type of data)	Dataset Size (Train/ Valid/Test)	Labeling Procedure	Augmentations	Deep learning algorithms	Hyperparameters	Hardware	Performance measures	Outcome
1	Aubreville M. et al., 2017 [19]	Germany	Confocal Laser Endomicroscopy images (OSCC)	7894 images	N/A	arbitrarily, randomly rotated copies	LeNet-5 with Transfer learning	3000 epochs Learning rate = 0.01 Optimizer: Adam	N/A	Accuracy Sensitivity Specificity AUC	88.3% 0.87 0.9 0.96
2	Ariji Y. et al., 2018 [20]	Japan	CT image of cervical lymph node (OSCC)	441 images	Annotated by a radiologist	altering the brightness, contrast, rotation, and sharpness	AlexNet	150 epochs	Nvidia GeForce GTX GPU workstation (Nvidea Corp., Santa Clara, CA, USA) with 11GB of memory	Accuracy Sensitivity Specificity PPV NPV AUC	78.2% 0.75 0.81 79.9% 77.1% 0.80
3	Xu S. et al., 2019 [21]	China	CT images (Oral cancer)	7000 images	Annotated by oral oncologist and a radiologist.	translational rotation and mirroring	LeNet-5	Learning rate = 0.1–0.01	N/A	Accuracy Sensitivity Specificity AUC	75.4% 0.82 0.74 79.6%
4	Ariji Y. et al., 2019 [22]	Japan	CT images (OSCC)	703 images (80% training and 20% test dataset)	Annotated by a radiologist	N/A	AlexNet	300 epochs	GeForce GTX 1080 Ti, NVIDIA with 11 GB of GPU, 128 GB of memory, and the open-source operating system Ubuntu OS v. 16.04.2	Accuracy Sensitivity Specificity PPV NPV	84.0% 0.67 0.9 69.2% 89.0%
5	Panigrahi S., Swarnkar T., 2019 [23]	India	Histopathological images (Malignant, benign)	386 images	N/A	rotating, inverting, and flipping	CNN	100 epochs	Ubuntu 16.04 and accelerated by a graphicprocessing unit (NVIDIA GeForce 6GTX 1080 T7i with 4X 32 GB of memory)	Accuracy	96.8%
6	Jeyaraj P.R. et al., 2019 [24]	India	Hyperspectral images (Oral cancer)	2400 images	N/A	N/A	ResNet	Momentum rate = 0.1 Learning rate = 0.5 Dropout rate = 0.25 Batch size = 75	Intel Xeon processors, 5.2 GHz and a GPU - NVIDIA series	Accuracy Sensitivity Specificity	94.8% 0.98 0.97
7	Kiruthika S., Rahmath Nisha S., 2020 [25]	India	Histopathological images (OSCC)	1224 images	N/A	N/A	CNN	N/A	N/A	Sensitivity Specificity Precision Recall	0.99 0.94 94.6% 99.5%
8	Ramalingam A. et al., 2020 [26]	India	Histopathological images (OSCC)	350 images (275 training, 75, and testing images)	N/A	N/A	- Inception- v3 - ResNet50	N/A	N/A	Accuracy	92.1%
9	Chinnaiyan R. et al., 2020 [27]	India	Histopathological images (OSCC)	696 images	N/A	N/A	CNN with Transfer learning	5 or more epochs	N/A	Precision Recall F1-score	92.0% 89.0% 91.0%
10	Heidari A. E. et al., 2020 [28]	USA	Optical coherence tomography (OSCC)	54 images (33 training, 21 validation, and test images))	N/A	N/A	AlexNet	120 iterations	GPU (Nvida GTX 1080),	Sensitivity Specificity	1.0 0.7
11	Das N. et al., 2020 [29]	India	Histopathological images (OSCC)	156 images	N/A	rotating, shearing, translation, zooming and flipping	- AlexNet, - VGG-16 - VGG-19 - Resnet-50 - CNN	50 epochs Learning rate = 0.0001 Optimizer: Adam	GPU based system under Linux operating system having Intel®Corei7® 8750 h processor with 16GB memory and GTX® 1060 graphics	Accuracy	96.6%
12	Fu Q. et al., 2020 [30]	China	Clinical oral images (OSCC)	6176 images (5775 training, and 401 validation images)	N/A	scaling, rotation, horizontal flipping and adjustment of the saturation and exposure	Deep learning algorithm	N/A	N/A	Sensitivity Specificity AUC	0.95 0.89 0.98
13	Musulin J. et al., 2021 [31]	Croatia	Histopathological images (OSCC)	322 images	N/A	horizontal flip, vertical flip, rotation	- InceptionV3 - InceptionResNetv2 - DenseNet201 - NASNet EfficientNetB3	Learning rate = 0.001–0.0001	N/A	AUC	0.95
14	Alosaimi W. et al., 2021 [32]	Saudi Arabia	Histopathological images (OSCC)	1224 images	N/A	scaling, cropping, flipping, padding, rotation, translation, affine transformation, brightness, contrast and saturation	- LeNet-5 - AlexNet - VGG - Inception - ResNet50	10,000 iterations Learning rate = 0.001 Batch size = 64	N/A	Precision Recall F1-score Accuracy	98.0% 99.0% 98.0% 98.0%
15	Tomita H. et al., 2021 [33]	Japan	CT images (OSCC)	320 images (224 training, 32 validation, and 64 test images))	N/A	horizontal flip, vertical flip, width shift, and height shift.	- Deep learning	N/A	N/A	Accuracy Sensitivity Specificity	90.9% 0.73 1.0
16	Carmalan S. et al., 2021 [34]	USA	Clinical oral images (OPMDs)	54 images (85:15 for Training and validation)	Annotated by clinical team members	horizontal flip, vertical flip	Transfer - learning on Inception-ResNet-V2	20 epochs Learning rate = 0.0003 Batch size = 64	N/A	Precision Recall F1-score Accuracy	99.3% 100.0% 97.9% 90.9%
17	Musulin J. et al., 2021 [35]	Croatia	Histopathological images (OSCC)	322 histology images	N/A	rotation, horizontal flip and vertical flip	- ResNet50 - ResNet101 - Xception - MobileNetv2	Learning rate = 0.001–0.000001 Optimizer: Bayesian	two Intel Xeon Gold CPUs (24 C/48 T, at 2.4 GHz), 768 GB of ECC DDR4 RAM, and five Nvidia Quadro RTX 6000 GPUs, with 24 GB of RAM, 4608 CUDA and 576 Tensor cores.	AUCmacro AUCmicro	0.96 0.03
18	Warin K. et al., 2021 [36]	Thailand	Clinical oral images (OSCC)	700 images (70:10:20 for training, validation, and test)	Annotated by three oral and maxillofacial surgeons	scaling, rotation, horizontal flipping, and adjustment of the saturation and exposure	- DenseNet121	N/A	2 of GPU, TitanXP 12GB, Nvidia Driver: 450.102 and CUDA: 11.0.	Precision Recall F1-score Sensitivity Specificity AUC	100.0% 99.0% 99.0% 0.99 1.0 0.99
19	Kavyashree C. et al., 2022 [37]	India	Histopathological images (OSCC)	526 images (70:15:15 for training, validation, and testing)	N/A	N/A	- CNN - DenseNet201 - DenseNet121 - DenseNet169	50 epochs Learning rate = 0.0001 Loss function: Binary Crossentropy	N/A	Precision Recall F1-score Accuracy TPR FPR	98.9% 98.9% 93.2% 85.0% 0.93 0.14
20	Arujuaid A. et al., 2022 [38]	USA	Histopathological images (OSCC)	448 images	Annotated by oral pathologists	N/A	- GoogLeNet - InceptionV3 - Transfer learning	N/A	N/A	Precision Recall F1-score Accuracy	90.0% 95.5% 92.8% 92.5%
21	Krishna S. et al., 2022 [39]	India	Histopathological images (OSCC)	1224 images	N/A	N/A	- CNN - VGG16 - ResNet50 - Ensemble - Learning (VGG16+ ResNet50)	N/A	N/A	Accuracy	62.50%
22	Sharma D. et al., 2022 [40]	India	Clinical oral images (OSCC)	329 images (70:10:20 for training, validation, and test)	N/A	flipping, zooming, and rotation	- VGG19 - VGG16 - MobileNet - InceptionV3 - ResNet50	50 epochs Batch size = 16 Learning rate = 0.001	Tesla 1xK80 graphics card	Precision Recall F1-score Accuracy	60.0% 43.0% 50.0% 76.0%
23	Shetty SK. et al., 2022 [41]	India	Histopathological images (OSCC)	1224 images (70:30 for training, and test)	N/A	N/A	- VGG16 - Inception V3 - ResNet50 - duck pack optimization with deep transfer learning	N/A	Intel Core i5 processor and 8 GB of RAM	Precision Recall F1-score Accuracy	95.5% 97.5% 96.4% 97.3%
24	Jubair F. et al., 2022 [42]	Jordan	Clinical oral images (OSCC, OPMDs)	716 images (79:7:14 for training, validation, and test)	N/A	N/A	- EfficientNet-B0 - VGG19 - ResNet101	Batch size = 32 Learning rate = 0.0001 Optimizer: Adam	N/A	Accuracy Sensitivity Specificity AUC	85.0% 0.87 0.85 0.93
25	Warin K. et al., 2022 [43]	Thailand	Clinical oral images (OPMDs)	600 images (70:10:20 for training, validation, and test)	Annotated by three oral and maxillofacial surgeons	N/A	- DenseNet-121 - ResNet-50	100 epochs Batch size = 32 Learning rate = 0.00001	Tesla P100, Nvidia driver: 460.32 and CUDA: 11.2 (Nvidia Corporation, CA, USA)	Precision Recall F1-score Sensitivity Specificity AUC	92.0% 98.0% 95.0% 0.98 0.92 0.95
26	Xu Z. et al., 2022 [44]	China	Histopathological images (OSCC)	757 images	N/A	N/A	- EfficientNet b0 - ShuffleNetV2 - ResNeXt_18	80 epochs Batch size = 80 Learning rate = 0.0005 Optimizer: Adam	Four NVIDIA Tesla K80 graphics cards	Accuracy AUC	98.1% 0.99
27	Fati S. M. et al., 2022 [45]	Saudi Arabia	Histopathological images (OSCC)	5192 images	N/A	multiangle rotation, flipping and shifting	- AlexNet - ResNet-18	28 and 33 epochs	N/A	Precision Recall Accuracy Sensitivity Specificity AUC	99.7% 99.0% 99.1% 0.99 0.99 0.99
28	Warin K. et al., 2022 [46]	Thailand	Clinical oral images (OSCC, OPMDs)	980 images (70:10:20 for training, validation, and test)	Annotated by three oral and maxillofacial surgeons	N/A	- DenseNet-169 - ResNet-101 - SqueezeNet - Swin-S	43, 100 epochs Batch size = 16, 32 Learning rate = 0.00001	Tesla P100, Nvidia driver: 460.32 and CUDA: 11.2 (Nvidia Corporation, CA, USA)	Precision Recall F1-score Sensitivity Specificity AUC	98.0% 99.0% 98.0% 0.99 0.99 1.0
29	Deif M. A. et al., 2022 [47]	Egypt	Histopathological images (OSCC)	1224 images (80:20 for training, and test)	N/A	N/A	- VGG16 - AlexNet - ResNet50 - Inception V3	Batch size = 32 Learning rate = 0.001	N/A	Precision Accuracy Sensitivity	96.3% 96.3% 0.99
30	Yuan W. et al., 2022 [48]	China	Optical Coherence Tomography images (OSCC)	468 images (346 training, and 122 test images)	Annotated by two senior dental specialists with professional diagnoses	N/A	Multi-Level - Deep Residual Learning	20 epochs	Nvidia Geforce 2080Ti	Accuracy Sensitivity Specificity PPV NPV AUC	87.5% 0.91 0.88 85.3% 90.2% 0.92
31	Yang S.Y. et al., 2022 [49]	China	Histopathological images (OSCC)	2025 images (1925 training, and 100 test images)	Annotated by senior pathologists	N/A	- Deep learning	80, 100 epochs Batch size = 64 Learning rate = 0.001 Optimizer: Adam Loss function: cross entropy	NVIDIA RTX 2080Ti (Abadi 2016)	Sensitivity Specificity F1-score PPV NPV	0.98 0.92 95.1% 82.4% 97.8%
32	Chang X. et al., 2023 [50]	China	Raman spectroscopy (OSCC)	16,200 Raman spectra	N/A	N/A	- AlexNet - VGGNet - ResNet50 - MobileNetV2 - Transformer	Batch size = 64 Learning rate = 0.0001 Optimizer: Adam	NVIDIA GeForce GTX 1080 Ti	Precision Recall Accuracy	92.3% 92.9% 92.8%
33	Afify HM. et al., 2023 [51]	Egypt	Histopathological images (OSCC)	1224 images	N/A	random, reflection, translation, resizing and rotation	- ResNet-101 - GoogleNet - SqueezeNet - ShuffleNet - AlexNet - DenseNet-201 - InceptionResNet-V2 - EfficientNet-b0 - VGG-19 - NasNetMobile with transfer learning methods	100 epochs Batch size = 15 Learning rate = 0.001 5200 and 5900 iterations	N/A	Precision Recall F1-score Accuracy Sensitivity Specificity	100.0% 100.0% 100.0% 100.0% 1.0 1.0
34	Agarwal P. et al., 2023 [52]	India	CT images (OSCC)	1755 images	Annotated by radiologists	horizontal flip, vertical flip shear and zoom	- BID-Net - VGG16 - VGG19 - ResNet-50 - MobileNetV2 - DenseNet-121 - ResNet-101	28 epochs Batch size = 15 Learning rate = 0.01, 0.001, 0.001 and 0.000 1	N/A	Precision Recall F1-score Accuracy AUC	91.0% 95.2% 92.6% 93.6% 95.9%
35	Oya K. et al., 2023 [53]	Japan	Histopathological images (OSCC)	90,059 images	N/A	horizontal flip, vertical flip, hue, saturation, contrast, brightness, cropping, rotation, zoom, and shift	EfficientNet B0	N/A	N/A	Precision Recall Accuracy	97.83% 98.36% 99.65%
36	Das M. et al., 2023 [54]	India	Histopathological images (OSCC)	1224 images (75:25 for training, and test)	N/A	Rotation, shift, zooming and shirring	- 10-layer CNN - VGG16 - VGG19 - Alexnet - ResNet50 - ResNet101 - Mobile Net - Inception Net	10, 50, 100 epochs Activation Function: ReLU Optimizer: Adam	N/A	Precision Recall F1-score Sensitivity Specificity Accuracy AUC Error rate	97.0% 98.0% 97.0% 0.98 0.97 97.0% 0.97 0.03
37	Flügge T. et al., 2023 [55]	Germany	Clinical oral images (OSCC)	1406 images (1124 training, 141 validation, and 141 test images)	N/A	N/A	Swin-Transformer	Learning rate = 0.005 Momentum = 0.9 Weight decay = 0.0001	12 GB NVIDIA TITAN V GPU	Accuracy F1-score Sensitivity Specificity PPV NPV	98.6% 98.6% 0.99 0.99 98.6% 98.6%
38	Ananthakrishnan B. et al., 2023 [56]	India	Histopathological images (OSCC)	1224 images	N/A	random rotation, translation and sheer	- ResNet50 - ResNet101 - ResNet152 - ResNet50V2 - ResNet101V2 - ResNet152V2 - Xception - VGG16 - VGG19 - InceptionV3 - InceptionResNetV2 - DenseNet201 - DenseNet121 - DenseNet169	N/A	NVIDIA Tesla K80	Sensitivity Specificity Accuracy AUC	99.3% 100.0% 99.7% 0.99
39	Panigrahi S. et al., 2023 [57]	India	Histopathological images (OSCC)	4000 images (2800 training, 400 validation, and 800 test images)	Annotated by pathologist	flipping, inverting, scaling, and rotation	- VGG16 - VGG19 - ResNet50 - InceptionV3 - MobileNet	Batch size = 32 Learning rate = 0.005 Momentum = 0.9 Weight decay = 0.0005 Optimizer: Adam	System (Quadro P5200) with a six-core i7 processor, 32 GB of GDDR5 RAM, and NVIDIA-2560 CUDA processing cores, 16 GB GPU (32 GB GDDR5 graphics memory and 2560 CUDA cores)	Precision Recall F1-score Accuracy	97.0% 96.0% 96.0% 96.6%
40	Yang Z. et al., 2023 [58]	China	Histopathological images (OSCC)	13,799 images (9737 training, and 4062 test images)	N/A	N/A	- LeNet-5 - VGG16 - ResNet18	40 epochs Batch size = 32 Learning rate = 0.0001 Momentum = 0.9 Optimizer: Adam	N/A	Precision Sensitivity Specificity Accuracy AUC	94.5% 99.5% 97.3% 96.8% 0.99

*PPV* Positive predict value, *NPV* Negative predict value, *TPR* True positive rate, *FPR* False positive rate, *AUC* Area under the curve

**Table 3:** Summary of findings in the selected diagnostic studies (object detection)

No	Author, Year (Ref)	Country	Data Modality (type of data)	Dataset Size (Train/ Valid/Test)	Labeling Procedure	Augmentations	Deep learning algorithms	Hyperparameters	Hardware	Performance measures	Outcome
1	Ariji Y. et al., 2020 [59]	Japan	CT images (OSCC)	365 images (Training: 260 images Validation: 60 images Test: 45 images)	Annotated by a radiologist	N/A	DetectNet	1000 epochs Learning rate 0.0001 Optimizer: Adam	graphic cards (GeForce GTX 1080 Ti, NVIDIA) with 11 GB of GPU and the opensource operating system Ubuntu OS v. 16.04.2.	Precision Recall F1-score	96.4% 73.0% 83.1%
2	Warin K. et al., 2021 [36]	Thailand	Clinical oral images (OSCC)	700 images (70:10:20 for training, validation, and test)	Annotated by three oral and maxillofacial surgeons	scaling, rotation, horizontal flipping, and adjustment of the saturation and exposure	Faster R-CNN	N/A	2 of GPU, TitanXP 12GB, Nvidia Driver: 450.102 and CUDA: 11.0.	Precision Recall F1-score AUC	76.7% 82.1% 79.3% 0.79
3	Warin K. et al., 2022 [43]	Thailand	Clinical oral images (OPMDs)	600 images (70:10:20 for training, validation, and test)	Annotated by three oral and maxillofacial surgeons	N/A	- Faster R-CNN - YOLOv4	100 epochs Batch size = 32 Learning rate = 0.00001	2 of GPU, TitanXP 12GB, Nvidia Driver: 450.102 and CUDA: 11.0.	Precision Recall F1-score AUC	79.7% 81.0% 80.3% 0.74
4	Warin K. et al., 2022 [46]	Thailand	Clinical oral images (OSCC, OPMDs)	980 images (70:10:20 for training, validation, and test)	Annotated by three oral and maxillofacial surgeons	N/A	- Faster R-CNN - YOLOv5 - RetinaNet - CenterNet2	1882 epochs Batch size = 8, 128 Learning rate = 0.001 15,000 and 20,000 iterations	Tesla P100, Nvidia driver: 460.32 and CUDA: 11.2 (Nvidia Corporation, CA, USA)	Precision Recall F1-score AUC	98.0% 92.0% 89.0% 0.91
5	Xu X. et al., 2023 [60]	China	CT images (OSCC)	5412 images (60:30:10 for training, validation, and testing)	Annotated by a radiologist	N/A	- Mask R-CNN	10, 50, 100 epochs	NVIDIA V100 GPU	AP50	72.5%

*AUC* Area under the curve

**Table 4:** Summary of findings in the selected diagnostic studies (segmentation)

No	Author, Year (Ref)	Country	Data Modality (type of data)	Dataset Size (Train/ Valid/Test)	Labeling Procedure	Augmentations	Deep learning algorithms	Hyperparameter	Hardware	Performance measures	Outcome
1	Das D.K., et al., 2019 [61]	India	Histopathological images (OSCC)	252 images (70:30 for training, and test)	N/A	N/A	CNN	50 epochs Learning rate = 0.01 Batch size 16	N/A	Dice index Jaccard index Precision Recall	94.2% 89.47% 97.6% 91.6%
2	Fraz M.M. et al., 2020 [62]	UK	Histopathological images (OSCC)	7780 images (5522 training, 1512 validation, and 756 test images)	Annotated by a pathologist	N/A	- FCN-8 - U-Net - Segnet - DeepLabV3+ - FABnet	50 epochs 45,000 iterations Learning rate = 0.0001 Batch size = 6	Nvidia GTX 1080Ti GPUs	Jaccard Index Dice index Accuracy Sensitivity Specificity Precision	78.4% 87.9% 96.3% 0.87 0.98 89.0%
3	Martino F. et al., 2020 [63]	Italy	Histopathological images (Oral cancer)	288 images (180 training, 100 validation, and 100 test image)	N/A	flipping the images vertically, horizontally, and in both ways	- SegNet. - U-Net - U-Net with VGG16 encoder. - U-Net with ResNet50 encoder	60 epochs Learning rate = 0.0001 Loss function: Cross-Entropy function	N/A	mIoU	0.63
4	Dos S. et al., 2021 [64]	Brazil	Histopathological images (OSCC)	1050 images (840 training, and 210 test image)	Annotated by a pathologist	horizontal/vertical flip, rotation, elastic transformation, grid distortion and optical distortion	Fully convolutional network	500 epochs Learning rate = 0.001 Batch size 16 Optimizer: Adam	Intel Core i7 3.4 GHz × 8 processor, 32 GB memory, 1 TB SSD) equipped with GeForce GTX 1050 Ti graphic card and Ubuntu 20.04 operational system	Accuracy Sensitivity Specificity F1 score Jaccard Index	97.6% 0.93 0.98 92.0% 85.2%
5	Paderno A. et al., 2021 [65]	Italy	Endoscopic videos (OSCC)	226 frames	Annotated by an expert clinician	rotation, shift, zoom, horizontal and vertical flip	- U-Net - U-Net 3 - ResNet	N/A	N/A	Dice index	76.0%
6	Musulin J. et al., 2021 [35]	Croatia	Histopathological images (OSCC)	322 histology images	N/A	Rotation, horizontal flip and vertical flip	DeepLabv3+ with Xception_65	Learning rate = 0.001–0.000001 Optimizer: Bayesian	two Intel Xeon Gold CPUs (24 C/48 T, at 2.4 GHz), 768 GB of ECC DDR4 RAM, and five Nvidia Quadro RTX 6000 GPUs, with 24 GB of RAM, 4608 CUDA and 576 Tensor cores.	mIoU F1 score	0.88 95.5%
7	Pennisi A. et al., 2022 [66]	Belgium	Histopathological images (OSCC)	389 WSI samples	Annotated by two pathologists	N/A	U-Net	N/A	N/A	Accuracy Dice index mIoU	82.0% 82.0% 0.72
8	Ariji Y. et al., 2022 [67]	Japan	CT images (OSCC)	983 images (834 training, 77 validation and 72 test image)	N/A	N/A	U-net	200 epochs Learning rate = 0.001	11 GB GPU (NVIDIA GeForce RTX 2080 Ti, NVIDIA, Santa Clara, CA, USA) and 32 GB of memory.	Precision Recall F1 score AUC	94.2% 74.2% 83.1% 95.0%
9	Liu Y. et al., 2022 [68]	USA	Histopathological images (Oral precancerous lesion)	39,264 images	Annotated by 112 pathologists	rotation, horizontal and vertical flips	- DeepLabv3+ - Unet++	20 epochs	Nvidia Titan GPUs	Accuracy Precision F1 score Sensitivity	90.9% 90.3% 93.3% 0.97
10	Dos S. et al. 2023 [69] (32)	Brazil	Histopathological images (OSCC)	200 histology images (100 training, and 100 test image)	N/A	rotation, transpose, and horizontal and vertical axis flipping	- Fully convolutional networks	400 epochs	Intel Core i7 3.4GHz × 8 processor, 32 GB memory, 1 TB SSD equipped with GeForce GTX 1050 Ti graphic card and Ubuntu 20.04 operational system	Accuracy Precision F1 score Sensitivity Specificity IoU	86.46% 76.63% 77.16% 0.81 0.91 0.63

*mIoU* Mean intersection over union, *AUC* Area under the curve

**Table 5:** Summary of findings in the selected prognostic prediction studies

No	Author, Year (Ref)	Country	Data Modality (Type of data)	Dataset Size	Inclusion Criteria (if any)	Exclusion Criteria (if any)	Hyperparameter	Augmentations	Deep learning algorithms	Hardware	Performance measures	Outcome
1	Kim D.W. et al., 2019 [70]	Republic of Korea	Clinicopathological data (OSCC)	255 patients’ records	N/A	patients with metastatic disease, secondary primary cancer, perioperative mortality, a history of previous radiotherapy or/and chemotherapy, or a history of previous head and neck cancer Patients with a follow-up period shorter than 36 months	N/A	N/A	- DeepSurv - Random survival forest (RSF) - Cox proportional hazard model (CPH)	N/A	c-index	0.78
2	Adeoye J. et al., 2021 [71]	Hong Kong	Clinicopathological and treatment data (OPMDs)	1098 patients’ records	minimum follow-up of 18 months	patients with synchronous erythroplakia and proliferative verrucous leukoplakia or those with previous oral cavity cancers	Batch size = 64, 128, 256 Drop out = 0.1–0.3 Nodes per layer = 32, 64, 128, 256 Optimizer: Adam Activation: ReLU	N/A	- DeepSurv - Neural net-extended time-dependent cox model (Cox-Time) DeepHit - RSF	N/A	c-index IBS	0.95 0.04
3	Adeoye J. et al., 2022 [72]	Hong Kong	Clinicopathological and treatment data (Oral cancer)	313 patients’ records	minimum follow-up period of 12 months	cases with carcinoma-in-situ, oral cancers with non-squamous histology, recurrent oral cavity tumors at first encounter, and patients with inoperable disease	Learning rate = 0.01, 0.001 Batch size = 64 Drop out = 0.4 Nodes per layer = 64	N/A	- DeepSurv - DeepHit - Cox-Time - RSF	N/A	c-index IBS	0.85 0.12

*c-index* Concordance index, *IBS* Integrated Brier score

**Fig. 2:**
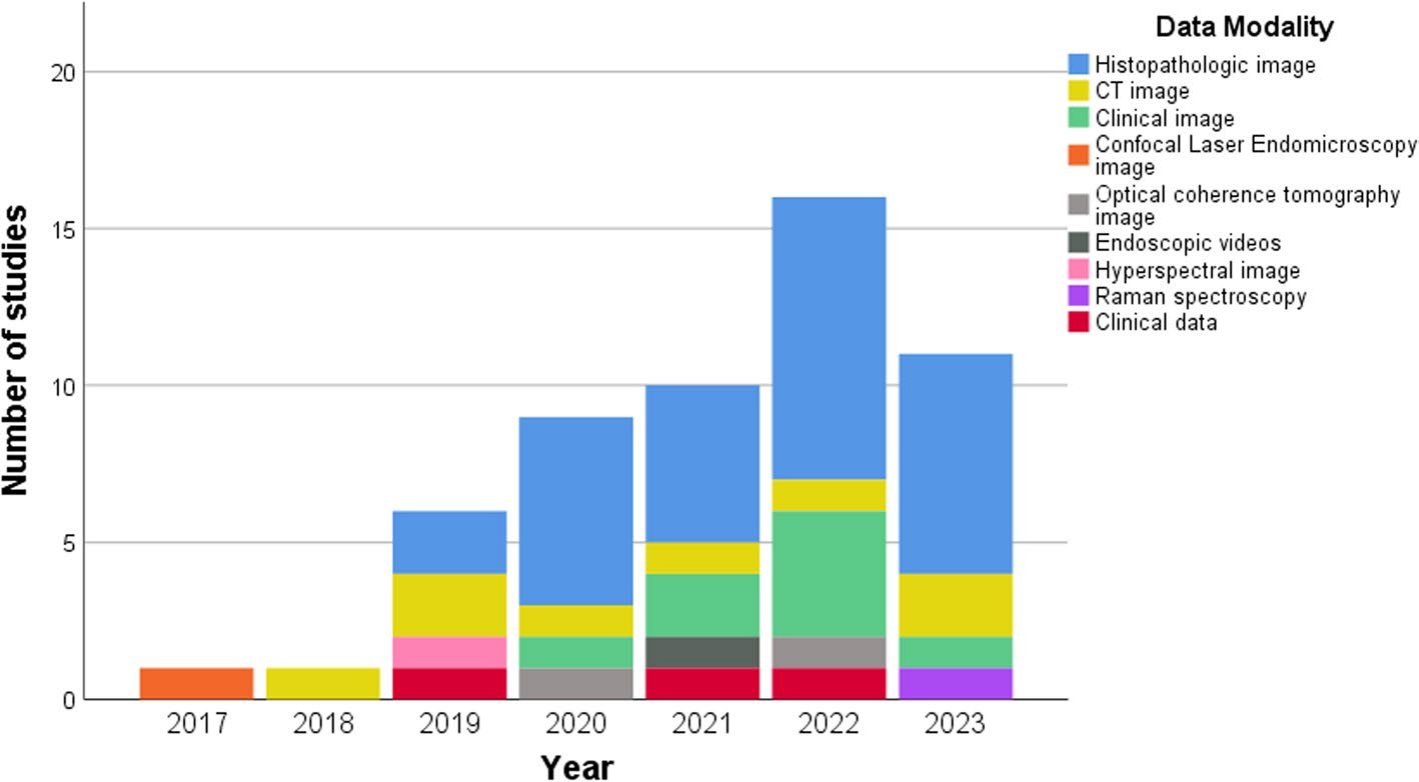
Number of DL studies for oral cancer and image type employed

Of the 54 included studies, 51 studies examined the use of DL applications in the diagnostic performance on medical images and 3 studies evaluated the prognostic prediction of DL applications. Most studies on the application of DL techniques in oral cancer were published recently, i.e. in 2019 and 2023 (*n* = 52) **(**Fig. 2**)**. With regards to the regions of relevant articles, 37 of the studies were carried out entirely in Asia, 9 in Europe, 2 in Africa and 6 in the United States.

Seven different types of imagery data were employed to the DL applications on diagnostic studies, including histopathological images (*n* = 30), CT images (*n* = 8), clinical oral images (*n* = 9), and other types of image (*n* = 4), including confocal laser endomicroscopy images, optical coherence tomography images, and endoscopic videos. Clinicopathological and treatment data (*n* = 3) were incorporated in the DL applications on prognostic prediction studies. In addition, types of oral cancer data which were used in the development of DL models included OSCC (*n* = 41), non-specific type of oral cancer (*n* = 5), OPMDs (*n* = 5), and multiclass analysis of OSCC and OPMDs (*n* = 3). In diagnostic studies, some studies used expert annotation to set the reference test (*n* = 19). Specifically, one human expert (*n* = 7), two (*n* = 3), three or more (*n* = 9) experts were involved in defining the reference test.

Regarding the DL task, the most often chosen task was classification (*n* = 40), followed by segmentation (*n* = 10) and object detection (*n* = 5). Various DL models were used. In classification studies, most of the studies used multiple DL models (*n* = 25), including transfer learning models and multi-layer perceptron, followed by customized CNN structures (*n* = 8), LeNet-5 (*n* = 2), AlexNet (*n* = 2), DenseNet121 (*n* = 1), EfficientNet B0 (*n* = 1), and Swin-Transformer (*n* = 1). Regarding segmentation, most of the studies used multiple DL models, including auto-encoders models (*n* = 5), customized CNN structures (*n* = 3), and single auto-encoders models (e.g., U-Net) (*n* = 2). Regarding object detection, one-stage object detectors (e.g., YOLO) or two-stage object detectors (e.g., Faster R-CNN) were used in the majority of studies (*n* = 5). Classification studies mainly reported on precision, recall (sensitivity), F1-score, accuracy, and specificity; other outcome measures were the area-under-the receiver-operating-characteristics curve (AUC)*.* In object detection studies, most studies were focused on precision, recall, F1-score, average precision (AP) and the AUC. Segmentation studies were more heterogeneous but additionally reported the Dice coefficient index and the mean Intersection over Union (mIoU). Furthermore, studies in prognostic prediction consistently reported the Concordance index (c-index) and Integrated Brier score (IBS) in all studies (Tables 3, 4 and 5).

### Risk of bias and applicability

Detailed information about modified leading questions of QUADAS-2 for critical appraisal and the risk of bias are presented in Table S1–S5. Among the included studies, 13 (24.1%) were found to have low risk of biases in all four domains. Moreover, 40 studies (74.1%) were evaluated as low risk for concerns regarding applicability. The most problematic domain was “Reference Standard”, where only 22 studies (40.7%) were classified as low risk of bias followed by “Patient selection” where 32 studies (59.3%) were classified as low risk of bias.

### Findings of the studies

In diagnostic studies, classification studies reported accuracies ranging from 85.0 to 100%, 78.2 to 93.62%, and 76.0 to 98.58% for classifying oral cancer on histopathological images, CT images and oral clinical images, respectively. The detection performance of object detection studies reported the F1-score ranging from 79.31 to 89.0%. In addition, the model performance of segmentation studies reported the Dice coefficient index ranging from 76.0 to 96.3%. In prognostic prediction studies, the prediction performance of DL models reported the c-index and IBS ranging from 0.78 to 0.95 and 0.04 to 0.12, respectively.

**Fig. 3:**
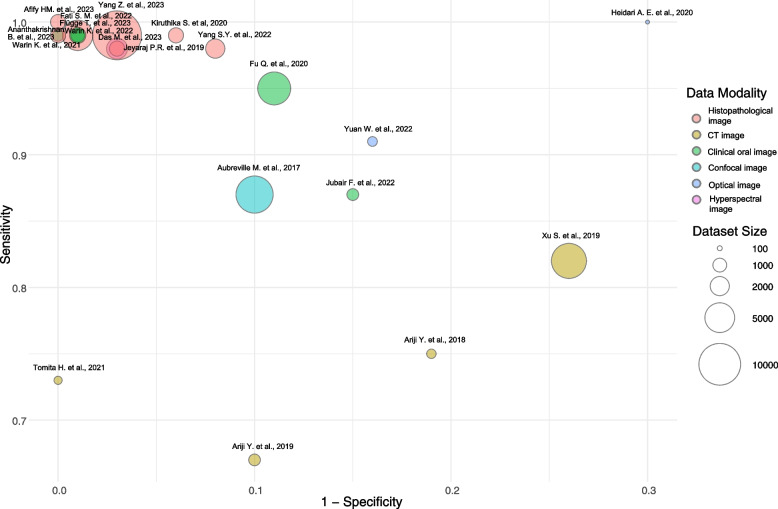
The various reported sensitivity and specificity of classification studies by sample size. The diameter of the circles represents the size of the dataset

**Fig. 4:**
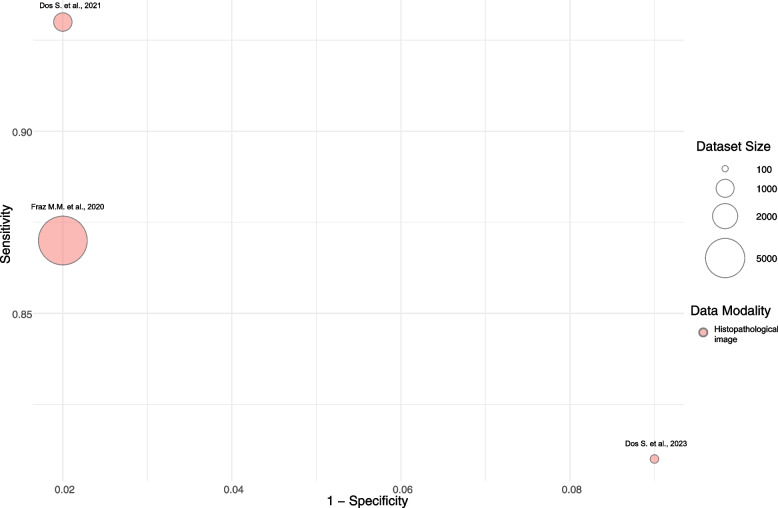
The various reported sensitivity and specificity of segmentation studies by sample size**.** The diameter of the circles represents the size of the dataset

As outlined, classification and segmentation studies of oral cancer were used for further synthesis. Of these, 23 studies could be pooled, including classification of 20 studies and segmentation of 3 studies. The pooled sensitivity, specificity, and DOR of classification studies were 0.92 (95% CI 0.87–0.97), 0.92 (95% CI 0.88–0.96), and 2549.08 (95% CI 410.77–4687.39), respectively **(**Fig. 3**)**. The pooled sensitivity, specificity, and DOR of segmentation studies were 0.87 (95% CI 0.72–1.02), 0.96 (95% CI 0.86–1.06), and 340.68 (95% CI -414.87 – 1096.22), respectively **(**Fig. 4**)**. In addition, the majority of studies used histopathological data to develop the DL-based image classification with a high sensitivity and specificity of 0.99 (95% CI 0.98–0.99), and 0.97 (95% CI 0.94–0.99), respectively.

## Discussion

Oral cancer is a life-threatening malignancy with frequent tumor metastasis and recurrence, which affects the survival rate and quality of life of patients [73–75]. The number of studies investigating the application of DL in oral cancer has increased in recent years. Most of the studies in this systematic review were published in 2019. This study compiled and assessed studies involving the DL for diagnosis and prognostic prediction of oral cancer by analyzing medical data including histopathological, CT, clinical image data, clinicopathological and treatment modality features data. Notably, however, the studies were of limited quality overall and comparison between studies was impeded by heterogeneity in conducting and reporting of the studies.

This systematic review found that most of the studies showed relatively high accuracy, sensitivity, and specificity of DL for the diagnosis of oral cancer (generally exceeding 80%). Nevertheless, heterogeneity in study conduct and reporting was high, precluding further comparisons between studies or quantitative synthesis. This review found that the included studies lacked details on the annotation process, did not mention the separation of the test dataset and the proportion between training, validation, and test dataset, which resulted in a high risk of bias in the reference test and patient selection. Additionally, seven diagnostic studies that mentioned the annotation process were annotated by one expert, resulting in these studies lacking inter-annotator agreement. To reduce the high risk of bias, future diagnostic studies should address minimum standard guidelines, such as Standards for Reporting of Diagnostic Accuracy Study-AI (STARD-AI); standards for diagnostic studies using AI models [76], Checklist for Artificial Intelligence in Medical Imaging (CLAIM); and a checklist for AI in medical imaging [77].

Regarding the heterogeneity in DL diagnostic studies of oral cancer, most studies did not report the value of TP, TN, FP, and FN; which caused a limitation for this systematic review of qualitative analysis of the results of oral cancer diagnostic study. Alternatively, the authors considered pooling sensitivity and specificity to calculate summary DORs as a single accuracy parameter. Moreover, the hyperparameter of DL models is essential for the explanation of tuning DL models to achieve the best performance from the model. This study found that several studies did not report the hyperparameters of DL models. This had a significant impact on the reliability and explainability of DL model performance, leading to a high risk of bias in the index test. To the best of our knowledge, there are no guidelines on reporting the hyperparameter tuning outcome/procedure for DL as models for medical diagnosis and prediction. This could explain why the hyperparameters reported in DL studies were heterogeneous.

Only three prognostic prediction studies applied DL algorithms, such as DeepSurv and DeepHit, in clinicopathologic and treatment modality data. The number of studies on DL was even less than studies in the era of machine learning (ML) [13, 14]. Nevertheless, the predictive performance of DL also yielded high accuracy for this task, achieving a c-index of 0.78–0.95 [70–72]. The predicted parameters were still the same as those of the ML era, which was interested in using clinicopathological and treatment modalities data to predict the prognosis and survival rate of oral cancer patients [13, 14]. Furthermore, there are no prognostic prediction studies of oral cancer in DL using molecular, cytological, and genomic data as a predictor, especially during preoperative evaluation. Combining various types of oral cancer data with the AI model could develop future prognostic prediction models allowing clinicians to decide on the most appropriate treatment plan to increase the survival rate of oral cancer patients.

All the studies included in this systematic review highlighted that DL techniques provide an increased precision approach for clinicians in making informed decisions. It should be emphasized that almost all the included studies only determined the accuracy performance of the DL model, in a few cases comparing it against the clinicians or experts. Furthermore, a fundamental element in achieving safe and efficient deployment of DL models in clinical practices is that the models achieve reliable generalizability. That is, the performance of the model when it is applied to external cases outside of the data for which it was trained [8, 10]. Therefore, the international collaboration among multiple healthcare centers could collect the data from multiple sources to develop the DL-based medical diagnosis and prognostic prediction system with the potential to be used in clinical practice. Nowadays, there are no standard guidelines for the appropriate accuracy of AI for clinical practice. Clinicians should understand that AI models are a decision support tool to improve treatment effectiveness and efficiency, but management options are based on the clinician’s decision.

This study has a number of strengths and limitations of the included studies and the review analysis. First, this review comprehensively and systematically appraised studies on DL for the diagnosis and prognostic prediction of oral cancer, and thus allows a narrative synthesis of the calculated DOR. Second, for limitation, this study selected only the scope of DL in oral cancer and found that studies reported heterogeneity, including various types of data and different reported outcome parameters, which was limited in qualitative analysis. In addition, this systematic review did not analyze the diagnostic performance of classification studies with the receiver operating characteristic (ROC) curve, which is one of the most widely used to analyze the diagnostic accuracy of classification models [78]. Future studies should critically determine reference tests and patient selection by addressing the checklist for AI in medical diagnostic and prognostic studies [76, 77, 79], which could improve utility to assess potential impact and clinical utility. Furthermore, many DL-based clinical image studies used image data from a public database and did not report diagnostic biopsy of lesions, which is an important ground truth that shows the reliability of the data for pathological AI research. Therefore, the future study should address the method to verify the reliability of clinical image from public database apart from biopsy proven to verify the ground truth of clinical image data for the medical AI study.

## Conclusions

This systematic review reveals the increasing number of DL studies in oral cancer with a diverse type of architectures. The reported accuracy showed promising performances for diagnostic and prognostic analyses in studies of oral cancer, Furthermore, this systematic review found that different oral cancer data modalities in diagnostic studies impacted the sensitivity and specificity results of DL. This presents researchers with opportunities to investigate DL algorithms to various data modalities. Finally, the application of DL in oral cancer appeared to have potential utility in improving informed clinical decision-making and providing better diagnosis and prognosis of oral cancer. Future work to improve the explainability and interpretability of DL models and the use of clinically applicable performance measures would be needed to translate these models for use in clinical practice.

### Supplementary Information

**Additional file 1: Table 1S**. Modified leading questions of QUADAS-2 for critical appraisal. **Table 2S**. Quality assessment of included studies using QUADAS-2 (Classification studies). **Table 3S**. Quality assessment of included studies using QUADAS-2 (Object detection studies). **Table 4S**. Quality assessment of included studies using QUADAS-2 (Segmentation studies). **Table 5S**. Quality assessment of included studies using QUADAS-2 (Prognosis prediction studies).

**Additional file 2.** PRISMA 2020 Checklist.

## Data Availability

The data of this study is available from the corresponding author upon reasonable request.
