# Supplementary material for: Deep learning in oral cancer- a systematic review
Source: BMC Oral Health. 2024 Feb 10;24:212. doi: 10.1186/s12903-024-03993-5 (PMC10859022; doi:10.1186/s12903-024-03993-5)
Supplement: Supplementary file 1 — Additional file 1: Table 1S. Modified leading questions of QUADAS-2 for critical appraisal. Table 2S. Quality assessment of included studies using QUADAS-2 (Classification studies). Table 3S. Quality assessment of included studies using QUADAS-2 (Object detection studies). Table 4S. Quality assessment of included studies using QUADAS-2 (Segmentation studies). Table 5S. Quality assessment of included studies using QUADAS-2 (Prognosis prediction studies). [file 12903_2024_3993_MOESM1_ESM.docx]

**Table 1S** Modified leading questions of QUADAS-2 for critical appraisal.

| **Domain** | **Leading Question** |
| --- | --- |
| Patient selection | 1. Were data imbalances (if there were any) addressed in the study?  2. Did the study avoid inappropriate data exclusion? (e.g., did not identify the diagnosis of image’s lesion)  3. Was a consecutive or random sample of patients (or data) enrolled?  4. Was the test dataset separate from the training and validation datasets?  **Applicability:** Are there concerns that the included data and setting did not match the review question? |
| Index test | 1. Were the deep learning method results interpreted without knowledge of the results of the reference standard?  2. If a threshold was used, was it pre-specified?  3. Was the method described in sufficient detail to reproduce the presented results?  **Applicability:** Are there concerns that the method, its conduct, or interpretation differed from the review question? |
| Reference standard | 1. Were the reference standard results interpreted without knowledge of the results of the index test?  2. Did the study use a biopsy as a gold standard in clinical image data?  3. If not, was the annotation procedure described in the study and found to minimize bias?  4. Did the study sufficiently report their limitations, biases, or issues around generalizability?  **Applicability:** Are there concerns that the target condition as defined by the reference standard did not match the question? |
| Flow and timing | 1. Were all data included in the analysis?  2. Was there an appropriate interval between the index test and reference standard?  3. Did all data have a reference standard?  4. Did all data have the exact reference standard? |

**Table 2S** Quality assessment of included studies using QUADAS-2 (Classification studies)

| **Reference/year** | **Risk of bias** | | | | **Applicability concerns** | | |
| --- | --- | --- | --- | --- | --- | --- | --- |
|  | **Patient selection (1)** | **Index test (2)** | **Reference standard (3)** | **Flow and timing (4)** | **Patient selection (5)** | **Index test (6)** | **Reference standard (7)** |
| Aubreville M. et al., 2017 [19] | HR | LR | HR | LR | LR | LR | LR |
| Ariji Y. et al., 2018 [20] | HR | LR | LR | LR | LR | LR | LR |
| Xu S. et al., 2019 [21] | HR | LR | LR | LR | LR | LR | LR |
| Ariji Y. et al., 2019 [22] | LR | LR | LR | LR | LR | LR | LR |
| Panigrahi S., Swarnkar T., 2019 [23] | HR | LR | HR | LR | LR | LR | LR |
| Jeyaraj P.R. et al., 2019 [24] | HR | LR | HR | LR | LR | LR | LR |
| Kiruthika S., Rahmath Nisha S., 2020 [25] | HR | HR | HR | LR | LR | HR | LR |
| Ramalingam A. et al., 2020 [26] | LR | HR | HR | LR | LR | HR | LR |
| Chinnaiyan R. et al., 2020 [27] | HR | LR | HR | LR | LR | LR | LR |
| Heidari A. E. et al., 2020 [28] | LR | LR | HR | LR | LR | LR | LR |
| Das N. et al., 2020 [29] | HR | LR | HR | LR | LR | LR | LR |
| Fu Q. et al., 2020 [30] | LR | HR | HR | LR | LR | HR | LR |
| Musulin J. et al., 2021 [31] | HR | LR | HR | LR | LR | LR | LR |
| Alosaimi W. et al., 2021 [32] | HR | LR | HR | LR | LR | LR | LR |
| Tomita H. et al., 2021 [33] | LR | HR | HR | LR | LR | HR | LR |
| Carmalan S. et al., 2021 [34] | LR | LR | LR | LR | LR | LR | LR |
| Musulin J. et al., 2021 [35] | HR | LR | HR | LR | LR | LR | LR |
| Warin K. et al., 2021 [36] | LR | HR | LR | LR | LR | HR | LR |
| Kavyashree C. et al., 2022 [37] | LR | LR | HR | LR | LR | LR | LR |
| Arujuaid A. et al., 2022 [38] | HR | HR | HR | LR | LR | HR | LR |
| Krishna S. et al., 2022 [39] | HR | HR | HR | LR | LR | HR | HR |
| Sharma D. et al., 2022 [40] | LR | LR | HR | LR | LR | LR | LR |
| Shetty SK. et al., 2022 [41] | LR | HR | HR | LR | LR | LR | LR |
| Jubair F. et al., 2022 [42] | LR | LR | HR | LR | LR | LR | LR |
| Warin K. et al., 2022 [43] | LR | LR | LR | LR | LR | LR | LR |
| Xu Z. et al., 2022 [44] | HR | LR | HR | LR | LR | LR | LR |
| Fati S. M. et al., 2022 [45] | HR | LR | HR | LR | LR | LR | LR |
| Warin K. et al., 2022 [46] | LR | LR | LR | LR | LR | LR | LR |
| Deif M. A. et al., 2022 [47] | LR | LR | HR | LR | LR | LR | LR |
| Yuan W. et al., 2022 [48] | LR | LR | LR | LR | LR | LR | LR |
| Yang S.Y. et al., 2022 [49] | LR | LR | LR | LR | LR | LR | LR |
| Chang X. et al., 2023 [50] | LR | LR | HR | LR | LR | LR | LR |
| Afify HM. et al., 2023 [51] | HR | LR | HR | LR | LR | LR | LR |
| Agarwal P. et al., 2023 [52] | HR | LR | LR | LR | LR | LR | LR |
| Oya K. et al., 2023 [53] | HR | HR | HR | LR | LR | HR | HR |
| Das M. et al., 2023 [54] | LR | LR | HR | LR | LR | LR | LR |
| Flügge T. et al., 2023 [55] | LR | LR | HR | LR | LR | LR | LR |
| Ananthakrishnan B. et al., 2023 [56] | HR | HR | HR | LR | LR | HR | HR |
| Panigrahi S. et al., 2023 [57] | LR | LR | LR | LR | LR | LR | LR |
| Yang Z. et al., 2023 [58] | LR | LR | HR | LR | LR | LR | LR |

LR, low risk; HR, high risk.

**Table 3S** Quality assessment of included studies using QUADAS-2 (Object detection studies)

| **Reference/year** | **Risk of bias** | | | | **Applicability concerns** | | |
| --- | --- | --- | --- | --- | --- | --- | --- |
|  | **Patient selection (1)** | **Index test (2)** | **Reference standard (3)** | **Flow and timing (4)** | **Patient selection (5)** | **Index test (6)** | **Reference standard (7)** |
| Ariji Y. et al., 2020 [59] | LR | LR | LR | LR | LR | LR | LR |
| Warin K. et al., 2021 [36] | LR | HR | LR | LR | LR | HR | LR |
| Warin K. et al., 2022 [43] | LR | LR | LR | LR | LR | LR | LR |
| Warin K. et al., 2022 [46] | LR | LR | LR | LR | LR | LR | LR |
| Xu X. et al., 2023 [60] | LR | LR | LR | LR | LR | LR | LR |

LR, low risk; HR, high risk.

**Table 4S** Quality assessment of included studies using QUADAS-2 (Segmentation studies)

| **Reference/year** | **Risk of bias** | | | | **Applicability concerns** | | |
| --- | --- | --- | --- | --- | --- | --- | --- |
|  | **Patient selection (1)** | **Index test (2)** | **Reference standard (3)** | **Flow and timing (4)** | **Patient selection (5)** | **Index test (6)** | **Reference standard (7)** |
| Das D.K., et al., 2019 [61] | LR | LR | HR | LR | LR | LR | LR |
| Fraz M.M. et al., 2020 [62] | LR | LR | LR | LR | LR | LR | LR |
| Martino F. et al, 2020 [63] | LR | LR | HR | LR | LR | LR | LR |
| Dos S. et al., 2021 [64] | LR | LR | LR | LR | LR | LR | LR |
| Paderno A. et al., 2021 [65] | HR | HR | LR | LR | LR | HR | LR |
| Musulin J. et al., 2021 [35] | HR | LR | HR | LR | LR | LR | LR |
| Pennisi A. et al., 2022 [66] | HR | HR | LR | LR | LR | HR | LR |
| Ariji Y. et al., 2022 [67] | LR | LR | HR | LR | LR | LR | LR |
| Liu Y. et al., 2022 [68] | HR | LR | LR | LR | LR | LR | LR |
| Dos S. et al. 2023 [69] | LR | LR | HR | LR | LR | LR | LR |

LR, low risk; HR, high risk.

**Table 5S** Quality assessment of included studies using QUADAS-2 (Prognosis prediction studies)

| **Reference/year** | **Risk of bias** | | | | **Applicability concerns** | | |
| --- | --- | --- | --- | --- | --- | --- | --- |
|  | **Patient selection (1)** | **Index test (2)** | **Reference standard (3)** | **Flow and timing (4)** | **Patient selection (5)** | **Index test (6)** | **Reference standard (7)** |
| Kim D.W. et al., 2019 [70] | LR | HR | LR | LR | LR | HR | LR |
| Adeoye J. et al., 2021 [71] | LR | LR | LR | LR | LR | LR | LR |
| Adeoye J. et al., 2022 [72] | LR | LR | LR | LR | LR | LR | LR |

LR, low risk; HR, high risk.
